# Immunoregulatory Effects of Elemental Diet and Its Ingredient, Tryptophan, via Activation of the Aryl Hydrocarbon Receptor in Mice

**DOI:** 10.3390/ijms25063448

**Published:** 2024-03-19

**Authors:** Atsuhito Kubota, Shungo Imai, Ryoichi Aoyagi, Wataru Murase, Masaru Terasaki, Mitsuru Sugawara, Yoh Takekuma, Hiroyuki Kojima

**Affiliations:** 1 School of Pharmaceutical Sciences, Health Sciences University of Hokkaido, 1757 Kanazawa, Ishikari-Tobetsu 061-0293, Hokkaido, Japan; atsuhito_k@hoku-iryo-u.ac.jp (A.K.); aoyagi.ryoichi.d2@elms.hokudai.ac.jp (R.A.); watarumurase@hoku-iryo-u.ac.jp (W.M.); terasaki@hoku-iryo-u.ac.jp (M.T.); 2 Department of Pharmacy, Hokkaido University Hospital, Sapporo 060-0817, Japan; msuga@pharm.hokudai.ac.jp (M.S.); y-kuma@pharm.hokudai.ac.jp (Y.T.); 3 Division of Drug Informatics, Faculty of Pharmacy, Keio University, Tokyo 108-0073, Japan; s-imai@keio.jp; 4 Advanced Research Promotion Center, Health Sciences University of Hokkaido, 1757 Kanazawa, Ishikari-Tobetsu 061-0293, Hokkaido, Japan; 5 Laboratory of Pharmacokinetics, Faculty of Pharmaceutical Sciences, Hokkaido University, Sapporo 060-0812, Japan

**Keywords:** inflammatory bowel disease, aryl hydrocarbon receptor, tryptophan, elemental diet

## Abstract

Inflammatory bowel disease (IBD) is characterized by chronic intestinal inflammation and its treatment varies widely; however, when inflammation is high, a complete nutrient containing pre-digested elemental diet (ED) is used to preserve the intestinal tract. In this study, we investigated the mechanisms underlying the effectiveness of EDs for IBD using mice. C57BL/6 mice were orally treated with the ED (5 mL/day) and its ingredient L-tryptophan (Trp) (1–100 mg/kg), respectively. Flow cytometry analysis revealed that treatment with the ED and Trp (10 and 100 mg/kg) significantly increased the percentage of splenic CD4+-/CD25+-/Foxp3+ regulatory T cells (Tregs). In the 2% DSS-induced colitis-mouse model, Trp administration (100 mg/kg) led to a significant decrease in TNF-α and increase in IL-10 in the serum as well as a significant decrease in the inflammation score. Furthermore, the aryl hydrocarbon receptor (AhR) agonistic activity, which is a key function of Treg induction, of Trp and 15 Trp metabolites was characterized using a highly sensitive DR-EcoScreen cell assay. Five Trp metabolites, including L-kynurenine, acted as AhR agonists, while Trp did not. Taken together, these results suggest that the ED treatment has a Trp-dependent immunoregulatory effect, and several Trp metabolites that activate the AhR might contribute to induction of remission in patients with IBD.

## 1. Introduction

Inflammatory bowel disease (IBD) is a general term used to describe chronic or remitting/relapsing inflammatory diseases in the intestinal tract. Ulcerative colitis (UC) is a diffuse, nonspecific inflammatory condition that presents as a continuous inflammatory lesion extending from the rectum, often forming erosions and ulcers [1]. Crohn’s disease (CD) is a chronic inflammatory disease characterized by discontinuous, full-layer granulomatous inflammation and fistulas that can involve any part of the gastrointestinal tract, from the oral cavity to the anus [2].

Various drugs, including 5-aminosalicylic acid (5-ASA) [3,4], immunosuppressive agents, and anti-tumor necrosis factor (TNF)-α antibody preparations, are used to treat IBD [5]. We have previously reported that 5-ASA, a key drug in IBD, induces the cluster of differentiation (CD)4+-/CD25+-/Foxp3+ regulatory T cells (Tregs) that suppress inflammation via the aryl hydrocarbon receptor (AhR), making it a potential new therapeutic target in the treatment of IBD [6]. The AhR is a ligand-activated transcription factor that regulates gene expression related to a wide variety of toxic and biological effects, such as birth defects, immunotoxicity, neurotoxicity, lethality, tumor promotion, and enzyme induction [7,8]. A novel study reported that a typical AhR activator, 2,3,7,8-tetrachlorodibenzo-p-dioxin (TCDD), affects the induction of CD4+ T cells, such as interleukin (IL)-17-producing T cells (Th17) and Tregs, via the activation of the AhR [9]. In recent studies on fields of inflammatory diseases, including cancer and autoimmune diseases, the AhR has been reported to act as an important molecule that regulates the differentiation of Tregs, which express CD25 and the transcription factor, forkhead box P3 (Foxp3) [10,11]. Furthermore, recent studies have shown that AhR activation induces production of an anti-inflammatory cytokine, IL-10 [12], and this has attracted attention as a novel therapeutic target for IBD [13].

Elemental diets (EDs) have been used over the last few decades for a variety of gastrointestinal disorders, including pancreatitis [14], to protect the intestinal tract. ED supplementation consists of an oral nutritional treatment that does not require digestion, and has an important role in the management of IBD patients, who commonly experience malnutrition [15]. However, the mechanisms underlying its effectiveness remain unknown. As EDs contains abundant L-tryptophan (Trp) (Table 1), which is metabolized by the gut microbiota and enzymes in vivo to enable it to bind and activate the AhR [16], it is possible that ED treatment for IBD may regulate inflammation through the induction of Tregs via AhR activation by Trp metabolites. In addition, EDs have been reported to enhance the effect of infliximab, an anti-TNF-α antibody [17], and to reduce the cumulative operative rate of CD [18], but the mechanisms of action in such cases remains unclear. 

In the present study, we sought to clarify the prescription rate of EDs among patients with IBD in Japan using the Claims Database and investigate the possible mechanisms underlying the effect of EDs on Tregs induction and intestinal inflammatory suppression in mice. In particular, we focus on Trp, which is an ingredient of EDs and is metabolized into several AhR activators [19,20]. In the two experiments using mice, we provide evidence that oral ED treatment (clinical dose) induces splenic Tregs as well as an increase of serum L-kynurenine (Kyn) concentration, and oral Trp administration in dextran sodium solution (DSS)-induced colitis model mice affect cytokine production, such as TNF-α and IL-10. In addition, we revealed in this study that Kyn might be a main mouse AhR activator in Trp-derived compounds. Namely, to date, although several Trp metabolites have been found to be AhR ligands from in vitro experiments, such as CYP1A1-induction, 7-ethoxyresorufin-O-deethylase (EROD), and transactivation assays, there are no comparative studies on AhR activity among a set of Trp metabolites using the same in vitro assay system. We previously developed a highly sensitive AhR-mediated transactivation assay that uses DR-EcoScreen cells [21], and reported that environmental chemicals, such as pesticides, plastic additives, and congeners of polychlorinated biphenyls, have AhR agonistic activity [21,22,23]. Therefore, our comparative data between Trp metabolites and AhR activity could be of great value in monitoring the lack of endogenous AhR ligands related to Tregs induction in inflammatory diseases, including IBD. Thus, here we provide some new insights into nutritional supplementation using ED, which not only prevents malnutrition but also possesses potential therapeutic effects via AhR-mediated Treg induction by several Trp metabolites.

## 2. Results

### 2.1. ED Prescribing Rates for IBD Patients Based on the JMDC Claims Database

**Figure 1:**
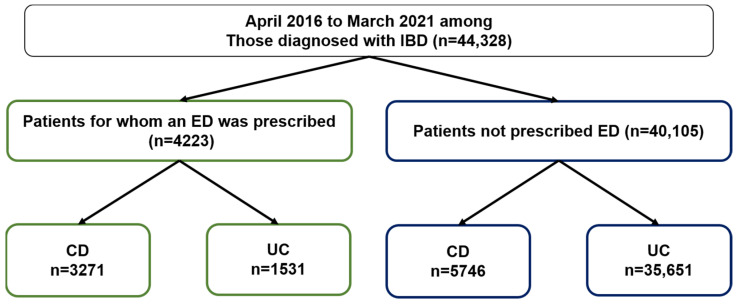
Number of patients diagnosed with IBD between April 2016 and March 2022 who were prescribed an ED, obtained from the JMDC Claims Database.

Japan has a universal health insurance system, in which all citizens have access to the same level of healthcare. We obtained data from the JMDC Claims Database for 44,328 patients diagnosed with IBD between April 2016 and March 2021. A total of 4223 patients (9.5%) were prescribed an ED. Of these, 3271 were diagnosed with CD and 1531 with UC (Figure 1).

### 2.2. Induction of Mouse Splenic Tregs by ED and Trp

**Figure 2:**
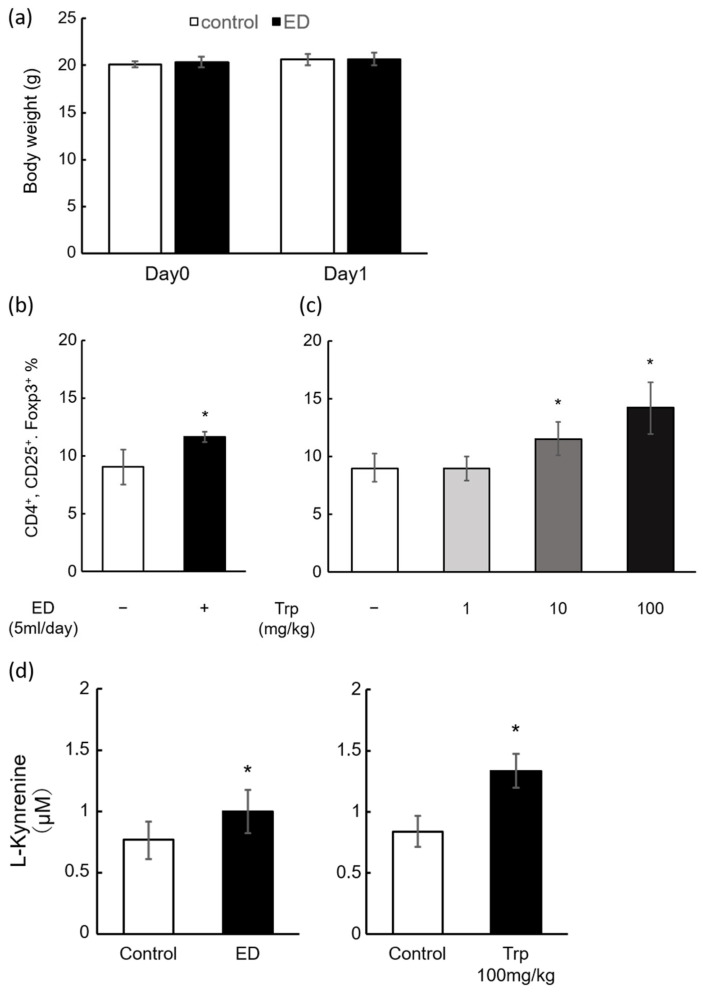
Effects of oral treatment with ED or its ingredient, Trp, on the induction of Treg differentiation in the splenocytes and serum Kyn concentrations in mice. C57BL/6 mice were orally treated with distilled water (vehicle control), 5 mL/day of ED and 1, 10, or 100 mg/kg of Trp, and sacrificed 24 h after administration. (**a**) Body weight (g) 24 h after administration. (**b**,**c**) Treg induction in each individual mouse spleen was measured by flow cytometry. (**d**) Serum Kyn concentrations (µM) were determined by LC-MS/MS 24 h after dosing. Values represent the means ± SD of five animals. * *p* < 0.05 vs. the vehicle control (leftmost bar), by (**a**,**b**,**d**) *t*-test, (**c**) Dunnet’s test.

Tregs are a group of T cells that suppress inflammation. C57BL6/N mice were exposed to a clinical dose of ED solution. After 24 h, there was no difference in body weight between them (Figure 2a), and ED significantly increased the number of mouse Tregs by approximately 1.3-fold (Figure 2b). The ED contains various components (Table 1), particularly Trp, the metabolites of which were formed by the gut microbiota in vivo and act as AhR agonists. We then administered Trp orally to mice using a sonde and observed a significant dose-dependent increase in splenic Tregs at 24 h (control: 9.0%, Trp 1 mg/kg: 8.9%, 10 mg/kg: 11.8%, 100 mg/kg: 15.1%, Figure 2c). Furthermore, LC-MS/MS (Liquid Chromatography-Tandem Mass Spectrometry) measurements of L-kynurenine (the main metabolite of Trp) in serum (Figure 2b,c) showed a significant increase in the ED administration and Trp treatment groups.

### 2.3. Effect of Oral Trp Administration on DSS-Induced Colitis Model Mice

**Figure 3:**
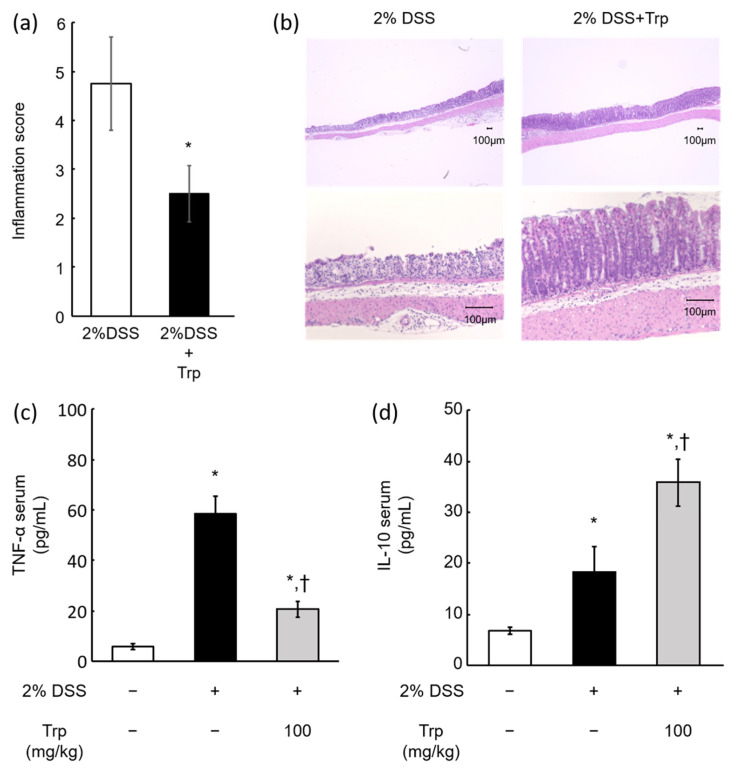
Inhibitory effects of Trp on inflammation in DSS-induced colitis model mice. (**a**) Inflammation score of the colonic mucosa, *; *p* < 0.05 by Dunnett’s test. (**b**) HE-stained image of the colon. (**c**,**d**) TNF-α and IL-10 ELISA results for mouse serum. *; vs. DSS, Trp double negative, *p* < 0.05 by Tukey’s test. †; vs. DSS (+), *p* < 0.05 by Tukey’s test.

The DSS-induced colitis model is a popular animal model of UC [24]. We created a colitis model in which animals were exposed to 2% DSS for 5 days and received 100 mg/kg aqueous solution Trp every 24 h during the DSS administration period. The inflammation score in the colon (4.75 ± 0.96 vs. 2.5 ± 0.58) was significantly reduced in the Trp group compared to that in the control group receiving the same amount of water (Figure 3a). In addition, hematoxylin and eosin (HE) staining showed that 2% DSS caused significant inflammation and villus loss in the mucosal epithelium of the colon. However, inflammation and villus loss were suppressed in the Trp group (Figure 3b). Furthermore, serum cytokines were evaluated by enzyme-linked immunosorbent assay (ELISA), with the results showing that DSS-induced TNF-α was significantly suppressed by Trp administration (DSS: 58.24 ± 6.98 pg/mL, DSS + Trp: 20.61 ± 3.21 pg/mL, Figure 3c). On the other hand, IL-10, a cytokine that suppresses inflammation, was significantly increased by Trp administration (DSS: 18.34 ± 4.88 pg/mL, DSS + Trp: 35.74 ± 4.59 pg/mL, Figure 3d).

### 2.4. AhR Agonist Activity of Trp Metabolites and In Silico Docking Analysis of Kyn, Tryptamine, or 3-Methylindole with AhR

**Figure 4:**
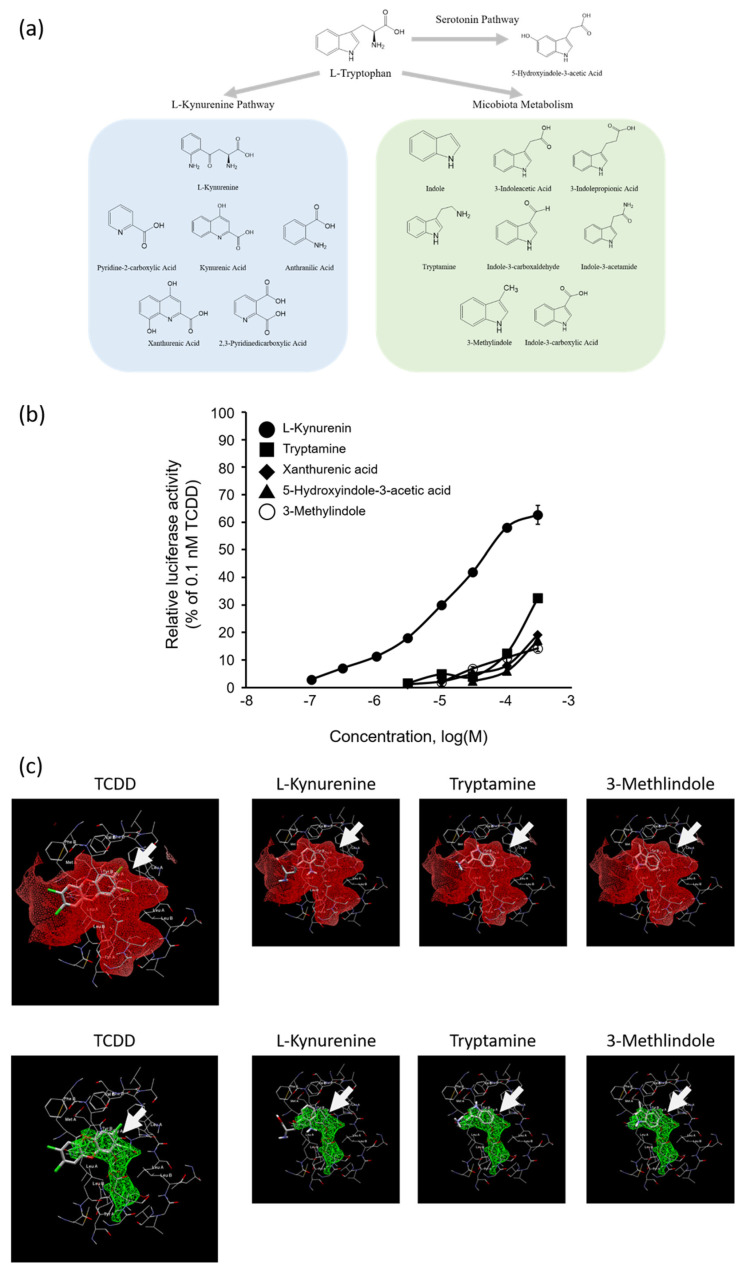
Effects of Trp metabolites on transcriptional activity via AhR and in silico docking analysis. (**a**) The metabolic pathways of tryptophan and Trp metabolites used in this study. (**b**) AhR-mediated transcriptional activity of active 5 Trp metabolites in DR-EcoScreen cell assay. The control was added at 0.1% DMSO, TCDD at 1 × 10^−10^ M, and other compounds at concentrations of ≤3 × 10^−4^ M. (**c**) Results of in silico docking simulation of TCDD, Kyn, tryptamine, and 3-methylindole against mouse AhR (PDB code 4M4X) using Molegro Virtual Docker. White arrows indicate docking sites.

Figure 4a shows the 15 Trp metabolites tested in this study. A transactivation assay using DR-EcoScreen cells, a mouse AhR reporter cell line, was undertaken to investigate the Trp and 15 Trp metabolites for AhR-mediated transcriptional activity. We found that, among the tested compounds, the five Trp metabolites of L-kynurenine (Kyn), xanthurenic acid, 5-hydroxyindole-3-acetic acid, 3-methylindole, and tryptamine had AhR agonistic activity greater than 10% of the TCDD-induced maximal activity, whereas Trp showed no significant difference compared with the control vehicle (Table 2). Figure 4b shows the dose-response curves for five compounds with AhR agonistic activity at concentrations of ≤3 × 10^−4^ M. The order of the relative AhR agonistic activity induced by the five compounds was Kyn >> tryptamine > 3-methylindole > xanthurenic acid > 5-hydroxyindole-3-acetic acid. The REC10 value for Kyn, the most potent AhR agonist in this study, was 7.0 × 10^−7^ M, which was 1.4 × 10^6^-fold higher than that of TCDD (REC10: 1.0 × 10^−12^ M) and about 100-fold lower than those of tryptamine and 3-methylindole (REC10: 6.3 × 10^−5^ M and 7.8 × 10^−5^ M, respectively) (Table 2). Trp and its 15 metabolites were not cytotoxic to the DR-EcoScreen cells at ≤3 × 10^−4^ M by the cell viability test with the Premix WST-1 Cell Proliferation Assay System. Figure 4c shows the results of in silico docking simulation of Kyn, tryptamine, and 3-methylindole against the AhR-ligand-binding domain. Analysis of these three ligand-binding sites using Molegro Virtual Docker showed that they all possessed affinities for ligand-binding sites similar to that of TCDD.

## 3. Discussion

While ED therapy has been used for the conservative treatment of the intestinal tract in the active phase of IBD, it has been clinically reported to have various secondary effects [15,17,18]. In particular, as certain amino acids, such as histidine, are decreased in patients with IBD [25], it has been hypothesized that EDs contribute to their replacement, and the anti-inflammatory effects of arginine have been reported [26]. However, the detailed mechanism of the anti-inflammatory effect by EDs remains unclear. Therefore, we focused on Trp, which is abundant in EDs, and conducted various evaluations. An older report suggested that EDs may not inhibit inflammation in CD and UC [15]. However, recent reports showed that EDs were effective for CD [27] and suggested that they were also effective, albeit to a limited extent, for UC [28]. In this study, approximately 10% of patients with IBD in Japan were found to have been prescribed EDs (Figure 1). This is the first report of its kind on the characteristics of IBD treatment in Japan on a large scale. In addition, ED prescription rates were 36% for CD and 4% for UC, suggesting that effectiveness of EDs is due to the abundance of evidence for CD. As there were a small number of cases in which ED was prescribed for UC, clarifying the mechanisms by which EDs regulate inflammation may lead to increase in the selection of EDs for the treatment of UC in the future.

After administration of clinical doses of ED (approximately 5 mL/day, ad libitum) to mice, Tregs in the spleen were significantly elevated (Figure 2a). Free contact with ED solution reduced water consumption and food intake, although no significant reduction in body weight was observed. No differences were observed in the inoculated calories or water intake when corrected for caloric units and calculated as ED intake plus water consumption. In addition, oral administration of Trp also showed a dose-dependent induction of Tregs (Figure 2b). These results indicate that ED treatment has Treg-inducing effects, and its ingredient, Trp, may contribute to the induction of Treg differentiation in the mouse spleen, suggesting that Treg induction by ED is mainly Trp-dependent. The 10–100 mg/kg used in this study is equivalent to 0.81–8.1 mg/kg in humans; the Trp in the ED is 151 mg/unit, which is 2.5 mg/kg (30.9 mg/kg in mice) on a standard body weight basis (60 kg) using one unit per day. The maximum clinical dose is approximately 7 units/day (human: 17.5 mg/kg, mouse: 218.75 mg/kg), which is equivalent to 3 units/day (human: 7.5 mg/kg, mouse: 92.3 mg/kg) for half ED therapy. ED is not palatable, and sometimes clinical cases are encountered where only 1/3 units can be taken. Therefore, our dose ranges were set at the lowest human dose (10 mg/kg) and a Trp dose (100 mg/kg) corresponding to half of the ED therapy. Furthermore, in an experiment using a mouse model of DSS-induced colitis, which is commonly used as an IBD model, the combination with Trp significantly suppressed the DSS-induced colitis (Figure 3a,b). Previous studies demonstrated that mice fed with a low-Trp diet became susceptible to chemically induced inflammation [29] and, conversely, mice or piglets fed with a Trp supplemented diet have reduced inflammation and a decreased severity of DSS-induced colitis [30,31]. These data, including our finding, suggest that a Trp-containing diet might ameliorate DSS-induced acute colitis and that Trp could serve as a promising preventive agent in the treatment of UC. A limitation of this study is that it was not possible to combine free contact with ED in a mouse model of DSS-induced colitis. This is because free contact with ED reduces the intake of aqueous DSS solution, which induces inflammation, making it difficult to create colitis. In our study, we focused on Trp and Trp metabolites to overcome these challenges and chose to administer the same amount of Trp using a sonde as the ED. In the present study, the behavior of TNF-α and IL-10 in the blood of a mouse model of DSS-induced colitis was similar to that previously reported [32,33,34]. In this case, a significant suppression in TNF-α and a significant increase in IL-10 were observed in the DSS + Trp group compared to the DSS group (Figure 3c,d). As IL-10 is reported to be produced by Tregs [12], the reason for the increase in IL-10 in the DSS + Treg group may have been the induction of Tregs by Trp. It has also been reported that compounds that modulate the metabolism of Trp by the gut microbiota induced IL-10 and suppressed inflammation in a mouse colitis model [35]. In addition, TNF-α is one of the most important cytokines involved in inflammation in IBD, and TNF-α inhibitors such as infliximab [36] and adalimumab [37] have shown high remission rates in both UC and CD. TNF-α is known to be negatively regulated by IL-10 [38], and the suppression of TNF-α was highly consistent with elevated IL-10. While the regulation of TNF-α, in particular, is extremely important in the treatment of IBD, treatment with antibody drugs has also been reported to decrease responsiveness due to the production of neutralizing antibodies [39]. Therefore, if Trp contained in EDs induces Tregs and suppresses TNF-α, it may provide a new option for IBD treatment. Tregs are a group of cells that are also known to play a major role in immune tolerance [40]. The induction of Tregs by the oral administration of EDs and Trp in the present study may explain the reduced rate of neutralizing antibodies produced by patients when EDs are combined with anti-TNF-α antibody drugs in clinical practice [39].

A lot of previous studies reported that Trp metabolites act as AhR agonists [19,20,33,41,42]. The primary Trp metabolism is known to be the kynurenine pathway that accounts for greater than 90% of the peripheral metabolism of Trp in mammals [43,44], and Trp is metabolized to Kyn via its metabolizing enzyme, indoleamine 2,3 dioxygenase (IDO)-1, which is reported to affect the Th17/Treg balance [32]. This increase in Kyn was reported to lead to an increase in Tregs [45]. In addition, microbial metabolism of Trp is also recognized as the source for the generation of a number of AhR ligands [46]. The 5-hydroxytryptamine pathway is a typical pathway for Trp. This pathway is initiated by tryptophan hydroxylase (THP)1,2. These products affect a variety of biological functions, including behavioral aspects (such as mood, libido, memory, appetite, and stress response), the central nervous system, gastrointestinal function, and platelet aggregation. Another important pathway is the kynurenine pathway; 95% of free Trp is a substrate for this pathway, and it is metabolized by indoleamine 2-3-dioxygenase (IDO)1,2 and tryptophan 2,3-dioxygenase (TDO) [47]. In this study, a DR-EcoScreen cell assay revealed that five of the fifteen Trp-derived compounds activated the AhR, while Trp itself had no AhR agonistic activity (Table 2, Figure 4b). The advantages of this study lie in the characterization of the order of AhR agonistic activity among Trp and its 15 metabolites under same assay system. To date, although Kyn, tryptamine, 3-methylindole, and xanthurenic acid were reported to be human and/or mouse AhR ligands [46], 5-hydroxyindole-3-acetic acid in serotonin pathway were newly identified as mouse AhR agonists. Noteworthy, the present study found that AhR agonistic activity of Kyn is extremely potent compared to those of other Trp metabolites. In addition, ED or Trp treatment significantly increased in mouse serum Kyn concentration (>1 × 10^−6^ M), showing AhR agonistic activity (REC_10_: 7 × 10^−7^ M) (Figure 2d and Figure 4b). Therefore, this finding indicates that splenic Treg induction and attenuation of DSS-induced colitis after administration of Trp may be due to AhR activation by Kyn converted from Trp. Additionally, in an in silico docking simulation of several Trp metabolites with AhR binding site, potent agonistic compounds (Kyn, tryptamine, and 3-methylindole) showed an affinity for the same binding site as TCDD, a typical ligand of AhR (Figure 4c), suggesting that these Trp metabolites activated AhR through binding to its ligand-binding site as well as TCDD did. The present study evaluated the agonistic activity of Trp metabolites against mouse AhR, and it has been reported that the ligand activity of several Trp metabolites is higher in evaluation systems using human AhR [48,49]. Thus, further study using human cell lines is required to examine the mechanisms underlying the effectiveness of ED therapy for patients with IBD. 

It has been reported that fecal AhR agonistic activities of IBD patients significantly decreased compared to those of healthy subjects [50]. In this study, our findings using mice indicate that the mechanism for Treg induction by Trp, which is abundant in EDs, involves in vivo metabolism by the intestinal microflora and conversion into compounds with AhR agonistic activity. The results of this experiment also indicate that EDs, which tend to be prescribed to approximately 10% of IBD patients in Japan, induce Tregs that suppress inflammation, and that the active ingredient may be Trp. Future analysis of serum TNF-α and IL-10 in patients clinically treated with EDs may further clarify the effects of EDs on humans.

## 4. Materials and Methods

### 4.1. Claims Database

Patient data were extracted from a nationwide health insurance Claims Database developed by JMDC Inc. (Tokyo, Japan). The database contains completely anonymized records of approximately 5.6 million insured persons from January 2005 to June 2021 (as of June 2022), who were primarily employed individuals and their family members under the age of 75 years, accounting for approximately 5% of Japan’s population. The records comprised information such as patient age and sex, and prescribed and/or dispensed drug names, doses, and prescription duration. Patients diagnosed with IBD between April 2016 and March 2021 (observation period) were selected; IBD diagnoses were classified using the International Classification of Diseases, 10th edition (ICD-10) codes. Medications were classified using ATC codes. 

### 4.2. Chemicals

Dimethyl sulfoxide (DMSO) and DMSO solution of TCDD (1 × 10^−7^ M) were purchased from Wako Pure Chemical Industries, Ltd. (Osaka, Japan). L-Kynurenine (>98% pure) were purchased from Tocris Bioscience (Bristol, UK). L-Tryptophan and Trp metabolites, except for L-kynurenine, were purchased from Tokyo Chemical Industry Co., Ltd. (Tokyo, Japan) and were greater than 98% pure. The True-NuclearTM One-Step Staining Mouse Treg FlowTM Kit was purchased from BioLegend (San Diego, CA, USA). All other reagents were of the highest grade available and were used as received without further purification. The ED used was Elental**^®^**, which is used in Japan, and its composition is shown in Figure 1. The product was purchased from Eisai Co., Ltd. (Tokyo, Japan).

### 4.3. Animals

Male C57BL/6 mice were obtained from Sankyo Laboratory Service (Shizuoka, Japan). Six-week-old mice weighing 18–22 g were housed in plastic cages under standard laboratory conditions of constant temperature (23 ± 2 °C) with a 12-h light-dark cycle and ad libitum access to water and food (standard rodent chow diet). All animal experiments were conducted according to the Guidelines for the Care and Use of Laboratory Animals of the Health Sciences University of Hokkaido.

### 4.4. Experimental Design

In the ED administration experiment, the ED group was provided free contact with water bottles filled with ED solution and sterile water for 24 h. In the Trp administration experiment, the control group was administered 10 mL/kg of water by forced oral gavage using a sonde tube. In the Trp group, 1–100 mg/10 mL/kg Trp was administered in the same manner, and the animals were euthanized after 24 h. The DSS-induced colitis protocol was the same as specified for BALB/c mice administered 2% DSS (MW 36,000–50,000 Da, Lot-No. S4140; MP Biomedicals, Santa Ana, CA, USA) in sterile water (*w*/*v*) for seven days, followed by 2% DSS for 5 days. The severity of colitis was measured using the DAI assessment, a scoring system that includes three parts: body weight loss (0–4), degree of intestinal bleeding (0–4), and stool consistency (0–4). During DSS administration, the control group received a forced oral dose of 10 mL/kg of water every 24 h using a sonde tube, while the Trp group received a similar dose of 1–100 mg/10 mL/kg Trp in an aqueous solution. All experiments were conducted with n = 4.

### 4.5. Flow Cytometry Analysis

Flow cytometry analysis was performed to determine Tregs present in mouse spleen cells. The spleens were harvested, crushed, washed with PBS (−), and centrifuged at 1000 rpm for 2 min, and the supernatant was discarded. All steps were performed according to the protocol of the True-Nuclear™ One-Step Staining Mouse Treg Flow™ Kit (BioLegend, San Diego, CA, USA). The cells were simultaneously stained with anti-CD4-PerCP, anti-CD25-PE, and anti-Foxp3-Alexa Fluor 488 antibodies (BioLegend, San Diego, CA, USA). Flow cytometry analysis was performed using a BD FACSaria IIIu flow cytometer (BD Biosciences, Franklin Lakes, NJ, USA). The resulting single cell populations were separated based on the fluorescence intensity of each antibody (Figure 3a). Tregs were identified as CD4+/CD25+/Foxp3+ cells. Data were analyzed using FACSDiva (BD Biosciences, USA) and FlowJo software v10.8.2 (Tree Star, Woodburn, OR, USA) [6,22].

### 4.6. Determination of L-Kyn in Mouse Serum

Measurement of L-kyn in mouse serum was subcontracted to NDTS Industries, Ltd. (Hokkaido, Japan) and was determined by LC-MS/MS. Blood samples were collected from the jugular vein after euthanasia by inhalation anesthesia according to NDTS protocols. Blood samples were centrifuged at 3000 rpm for 10 min, and 50 μL of the supernatant was collected.

### 4.7. Quantification of TNF-α and IL-10 in Mouse Serum Using Enzyme-Linked Immunosorbent Assay (ELISA)

The mouse serum TNF-α and IL-10 levels were measured using an ELISA kit (PGI Proteintech Group, Inc. Rosemont, IL, USA) according to the manufacturer’s instructions.

### 4.8. Histophathological Evaluation of the Intestines

Samples of the mouse colon were taken after reperfusion, and the internal portion was washed with phosphate-buffered saline (PBS) (−). Tissues were immediately fixed in 10% buffered formalin. Fixed tissue samples were then embedded in paraffin and sectioned. Slides were stained with HE to evaluate intestinal morphology and observed under a light microscope for classification [51].

### 4.9. AhR-Mediated Transactivation Assay Using DR-EcoScreen Cells

DR-EcoScreen cells were maintained in α-minimum essential medium (α-MEM) containing 5% fetal bovine serum (FBS), as previously described [6,22]. In brief, the cell suspension (1 × 10^5^ cells/mL) was seeded in a 96-well plate at 90 μL/well. After cultivation for 24 h at 37 °C, 10 μL of each of the various concentrations of Trp, its metabolites or TCDD dissolved in 1% DMSO were added to each well (the final concentration of DMSO was 0.1%). Following cultivation for another 24 h, 100 μL of Steady-Glo^TM^ reagent (Promega, Madison, WI, USA) was added to each well. The plate was then shaken at room temperature for 5 min, and luminescence was measured using a microplate luminometer (Wallac 1420 ARVOTM SX, Perkin-Elmer, Shelton, CT, USA). The results are expressed as mean ± SD from at least three independent experiments performed in triplicate [6,22].

### 4.10. Cell Viability Test and Evaluation of AhR Agonistic Activity

DR-EcoScreen cells were plated and cultured in the same way as the luciferase reporter gene assay, and WST-1 reagent was added 24 h after addition of test compounds. The cell viability was measured in accordance with the manufacturer’s protocol of Premix WST-1 Cell Proliferation Assay System (Takara, Kyoto, Japan). In order to estimate the potency of the AhR agonistic activity of the tested compounds, the luminescence intensity of the assayed compounds was presented as a dose-response curve. The concentration of the compound equal to 10% of the maximal response of TCDD was obtained from the dose-response curve of the luminescence intensity and expressed as the REC10 (10% relative effective concentration) value [22]. When the AhR agonistic activity of the test compound was higher than the REC10 value within the concentration tested (≤3 × 10^−4^ M), we judged the Trp and its metabolites to be positive for AhR agonistic activity. These values can be compared to that of TCDD.

### 4.11. Statistical Analysis

An analysis of variance (ANOVA) followed by Bonferroni correction was used to evaluate the differences in transcriptional levels between the control group and each of the other groups. The level of significance was set at *p* < 0.05. Data were represented as the mean ± S.D. of three triplicate experiments. Statistical significance in the mouse experiments was examined using ANOVA, Student’s *t*-test, Tukey’s test, or Dunnett’s test for post-hoc analysis. Differences were considered statistically significant at *p* < 0.05.

## 5. Conclusions

We clarified the Treg-inducing effect of EDs prescribed to 10% of patients with IBD in Japan. Animal experiments using a DSS-induced colitis-mouse model revealed that oral administration of Trp, abundantly contained in ED, attenuated inflammation, and its effective mechanism may involve a decrease in TNF-α and an increase in IL-10. AhR agonistic activity is a key function of Treg induction, with Trp and 15 Trp metabolites found in five Trp metabolites with the most potent compound, Kyn. Therefore, the immunoregulatory effects of EDs may be owing to several Trp metabolites (including Kyn) via AhR activation. This study provided evidence that EDs are useful for therapy in patients with IBD.

## Figures and Tables

**Table 1:** Composition of one bottle (80 g) of Elental^®^, an ED.

Composition	80 g/Unit
L-Isoleucine	642 mg
L-Leucine	899 mg
L-Lysine hydrochloride	888 mg
L-Methionine	648 mg
L-Phenylalanine	871 mg
L-Threonine	523 mg
L-Tryptophan	151 mg
L-Valine	701 mg
L-Histidine hydrochloride hydrate	501 mg
L-Arginine hydrochloride	1125 mg
L-Alanine	899 mg
L-magnesium and potassium aspartate	1036 mg
L-Aspartate sodium monohydrate	867 mg
L-Glutamine	1932 mg
Glycine	505 mg
L-Proline	630 mg
L-Serine	1159 mg
L-Tyrosine	110 mg
Dextrin	63.41 g
Sodium citrate hydrate	616 mg
Potassium chloride	150 mg
Calcium glycerophosphate	825 mg
Ferrous gluconate dehydrate	15.5 mg
Zing sulphate hydrate	7.88 mg
Manganese sulphate pentahydrate	1.30 mg
Copper sulphate	0.82 mg
Potassium indide	19.6 µg
Thiamine chloride hydrochloride	194 µg
Riboflavin phosphate sodium	256 µg
Pyridoxine hydrochloride	267 µg
Cyanocabalamin	0.7 µg
Calcium pantothenate	1.19 mg
Nicotinic acid amide	2.20 mg
Folic acid	44 µg
Biotin	39 µg
Choline bis (tartrate)	17.93 mg
Ascorbic acid	7.80 mg
Retinol acetate	648 IU
Tocopherol acetate	3.30 mg
Ergocalciferol	1.3 µg
Phytonadione	9 µg
Soybean oil	509 mg
total calories	300 kcal

**Table 2:** Comparison of AhR agonistic activity induced by Trp and its 15 metabolites.

Compound	REC_10_ ^(a)^	Relative Potency ^(b)^
TCDD	1.0 × 10^−12^	1
L-Tryptophan	N.E. ^(c)^	<3.3 × 10^−9^
L-Kynurenine	7.0 × 10^−7^	1.4 × 10^−6^
Tryptamine	6.3 × 10^−5^	1.6 × 10^−8^
3-Methylindole	7.8 × 10^−5^	1.3 × 10^−8^
Xanthurenic acid	1.2 × 10^−4^	8.3 × 10^−9^
5-Hydroxyindole-3-acetic acid	1.5 × 10^−4^	6.7 × 10^−9^
Indole-3-carboxylic acid	N.E.	<3.3 × 10^−9^
Indole-3-carboxaldehyde	N.E.	<3.3 × 10^−9^
Indole-3-acetamide	N.E.	<3.3 × 10^−9^
3-Indoleacetic acid	N.E.	<3.3 × 10^−9^
3-Indolepropionic acid	N.E.	<3.3 × 10^−9^
Indole	N.E.	<3.3 × 10^−9^
Kynurenic acid	N.E.	<3.3 × 10^−9^
Anthranilic acid	N.E.	<3.3 × 10^−9^
Pyridine-2-carboxylic acid	N.E.	<3.3 × 10^−9^
2,3-Pyridinedicarboxylic acid	N.E.	<3.3 × 10^−9^

^(a)^ 10% relative effective concentration (M): the concentration of the test compound showing 10% of the agonistic activity induced by 1 × 10^−10^ M TCDD. ^(b)^ Relative potency is expressed as a ratio of REC10 of TCDD to that of a test compound; i.e., the relative potency of TCDD is arbitrarily set at 1. ^(c)^ No effect (REC10 > 3 × 10^−4^ M).

## Data Availability

All data generated or analyzed during this study are available from the corresponding author on reasonable request.
